# A comparison of statistical emulation methodologies for multi‐wave calibration of environmental models

**DOI:** 10.1002/env.2405

**Published:** 2016-09-12

**Authors:** James M. Salter, Daniel Williamson

**Affiliations:** ^1^ College of Engineering, Mathematics and Physical SciencesUniversity of ExeterExeterU.K.

**Keywords:** history matching, uncertainty quantification, Gaussian processes, ensemble design, emulator diagnostics, tuning

## Abstract

Expensive computer codes, particularly those used for simulating environmental or geological processes, such as climate models, require calibration (sometimes called tuning). When calibrating expensive simulators using uncertainty quantification methods, it is usually necessary to use a statistical model called an emulator in place of the computer code when running the calibration algorithm. Though emulators based on Gaussian processes are typically many orders of magnitude faster to evaluate than the simulator they mimic, many applications have sought to speed up the computations by using regression‐only emulators within the calculations instead, arguing that the extra sophistication brought using the Gaussian process is not worth the extra computational power. This was the case for the analysis that produced the UK climate projections in 2009. In this paper, we compare the effectiveness of both emulation approaches upon a multi‐wave calibration framework that is becoming popular in the climate modeling community called “history matching.” We find that Gaussian processes offer significant benefits to the reduction of parametric uncertainty over regression‐only approaches. We find that in a multi‐wave experiment, a combination of regression‐only emulators initially, followed by Gaussian process emulators for refocussing experiments can be nearly as effective as using Gaussian processes throughout for a fraction of the computational cost. We also discover a number of design and emulator‐dependent features of the multi‐wave history matching approach that can cause apparent, yet premature, convergence of our estimates of parametric uncertainty. We compare these approaches to calibration in idealized examples and apply it to a well‐known geological reservoir model.

## 1 INTRODUCTION

Computer models are often used to represent physical systems, in order to study them under various scenarios or future events. These are based on using equations and algorithms to simulate physical processes, taking a given set of inputs and returning a representation of the physical system (for example, climate models as in Gordon *et al.* (2000); & Pope, Gallani, Rowntree, & Stratton (2000)). Inputs to a computer model vary in type and function, from those clearly representing real‐world processes or forcing (e.g., CO_2_ concentration in a climate model), to those without a direct physical analog, normally part of a “parametrization” of a process. The latter type of input parameter needs to be “tuned” so that the model represents the physical system it is intended to simulate as well as possible. Tuning of climate models is discussed in, for example, Mauritsen *et al.* (2012) and Hourdin *et al.* (2016).

Due to the complexity of these computer models and the lack of available computing time, we are unable to explore input parameter space through running the model at all parameter settings of interest (Santner, Williams, & Notz, 2003; Cumming & Goldstein, 2009). Instead, we usually design an ensemble of runs of the model and must use this in future analyses (Murphy *et al.*, 2004; Williamson, 2015). For complex models, such as climate models, it will rarely be the case that the full diverse range of potential model behaviors is adequately sampled by a single ensemble of runs.

The inability to run the computer model as often as we would like in many cases introduces uncertainty into any analysis and has an effect on any model‐based inferences we wish to make. Kennedy and O'Hagan (2001) outline the different types of uncertainty that must be accounted for. These can be quantified via the use of statistical models. For example, parametric uncertainty arises as we have unknown inputs for the model, and code uncertainty is introduced because we only have access to a limited number of runs of the model, and will not exactly know the output at other parameter settings.

In order to account for code uncertainty, we build statistical models called “emulators” (Sacks, Welch, Mitchell, & Wynn, 1989a; Currin, Mitchell, Morris, & Ylvisaker, 1991; Haylock & O'Hagan, 1996; Craig, Goldstein, Seheult, & Smith, 1996; Santner, Williams, & Notz, 2003) trained using the ensemble. An emulator is used to give a prediction of the computer model output at a given input *x*, along with an associated measure of the uncertainty on this prediction. The main reason to build emulators is that it is significantly more efficient to evaluate the output for a new parameter choice than if we were to run the computer model, although we may have considerable uncertainty on this output.

A common approach to emulation, particularly in application areas such as climate science, is to use regression‐only models (Rougier, Sexton, Murphy, & Stainforth, 2009; Sexton, Murphy, Collins, & Webb, 2011; Holden *et al.*, 2013; Williamson *et al.*, 2013; Williamson, Blaker, Hampton, & Salter, 2015), fitting a polynomial surface in the input parameters to the training data, without a correlated residual term. More common in the statistics literature is to use a Gaussian process to model the residual (Higdon, Nakhleh, Gattiker, & Williams, 2008a; Higdon, Gattiker, Williams, & Rightley, 2008b; Vernon, Goldstein, & Bower, 2010; Lee *et al.*, 2013; McNeall, Challenor, Gattiker, & Stone, 2013). This allows us to account for local variation away from the fitted polynomial surface, accounting for the fact that the residuals at physically close input parameter settings are likely to be positively correlated.

There are two main arguments for using regression‐only emulators, particularly in environmental modeling applications. The first is that there is a significant speed advantage in not having to form and invert the required covariance matrices during calibration‐type calculations (see Kaufman, Bingham, Habib, Heitmann, & Frieman, 2011 for discussion of the computational issues here). The second is that because there is unstructured uncertainty due to the initial conditions (internal variability) present when evaluating many environmental models, it is argued that the polynomial surface may capture “enough” of the model signal that the residual need not be correlated and can be used to represent this internal variability. In a sense, the argument is that it does not make enough of a difference to be worth the computational effort. The validity of this argument will depend on both the nature of the model and the proposed application of the emulator. In this paper, we explore the validity of this argument for emulators designed to assist in the calibration of environmental models.

We do this, not simply because of the popularity of regression‐only emulators for working with climate models, an important class of environmental models, but also because the majority of environmental models share a common feature that makes regression‐only modeling appealing, namely, an element of output uncertainty not due to code uncertainty (not specifically induced by the parameters and usually due to uncertainty in initial conditions). The temptation is to believe that because a correlation‐free residual term is required in modeling the code output as a function of the input parameters, the proportion of the residual from a regression fit that is correlated in the input parameters is insignificant (in that it will have relatively little effect on a calibration exercise).

In this paper, we will explore the impact of the decision to use different types of emulator for the calibration of simple toy models that share many of the features of environmental models. The calibration method we consider is known as “history matching (HM)” (Craig, Goldstein, Seheult, & Smith, 1996) and has been used in several climate applications (Edwards, Cameron, & Rougier, 2011; Gladstone *et al.*, 2012; Williamson *et al.*, 2013; McNeall, Challenor, Gattiker, & Stone, 2013; Williamson, Blaker, Hampton, & Salter, 2015). HM differs from Bayesian calibration (BC) (Kennedy & O'Hagan, 2001; Rougier, 2007; Higdon, Nakhleh, Gattiker, & Williams, 2008a; Sexton, Murphy, Collins, & Webb 2011) in that while BC looks to put a probability distribution on the setting of the input parameters that “best fits” observations, HM aims to refocus the search for reasonable models by removing regions of parameter space that are inconsistent with the observations from the search (Section 2.2).

In this article, we compare the popular approaches to emulation as they impact calibration. We use several illustrative examples and the commonly used borehole function in order to complete the comparative study and comment on some of the ensemble design issues that the decision to history match over multiple waves and the choice of emulator type at each wave raises. We then test our findings on a geological reservoir model, the IC fault model (Tavassoli, Carter, & King, 2004; Tavassoli, Carter, & King, 2005). Section 2 provides an overview of emulation and HM, with a focus on Gaussian process emulators, followed by a description of the experiments performed in this paper. Section 3 presents our simulation study using combinations of regression‐only and Gaussian process emulators for four examples. Section 4 applies the method to the IC fault model. Section 5 contains discussion. The appendices contain the example functions used in our study, present some of the technical details of our statistical modeling and emulator validation, and a short study of the impact of design and modeling judgments on calibration over multiple waves.

## 2 EMULATION AND HISTORY MATCHING

### 2.1 Emulation

Define the input space X of a computer model *f*, a vector‐valued function taking inputs x∈X. For output *i* of the computer model, the general form of an emulator is (Sacks, Schiller, & Welch, 1989b; Craig, Goldstein, Seheult, & Smith, 1996; Craig, Goldstein, Rougier, & Seheult, 2001)

(1) $$f_{i}(x)=\overset{k}{\underset{j=1}{\sum }}\beta_{\mathrm{ij}}h_{j}(x)+\varepsilon_{i}(x)+\nu_{i}(x)$$

where *h*_*j*_(*x*) are chosen functions of the parameters, *β*_*ij*_ are the unknown regression coefficients, *ε* is the systematic departure from the fitted linear model, assumed to be unknown and taken to be the realization of a Gaussian process with mean zero and a specified covariance function, and *ν* is the nugget, representing the additional variation in the response, with mean zero and the same variance for all *x*. The *β*s, *ε*, and *ν* are assumed to be uncorrelated.

Reasons for including a nugget term have been given by Andrianakis and Challenor (2012) and Gramacy and Lee (2012). Due to the internal variability of climate models (Hawkins & Sutton 2009), where different responses can be achieved for the same input parameters due to varying initial conditions, a nugget is needed when emulating climate model output (Williamson & Blaker 2014).

A Gaussian process is a stochastic process where the joint distribution of a finite number of random variables from this process is multivariate normal (Rasmussen & Williams 2006). It is completely defined by a mean function and a covariance function. There are many valid choices for covariance functions, usually denoted by *C*(·,·), where *C*(*x*, *x* ^*′*^) gives the covariance between the response at points *x* and *x* ^*′*^ in the input space. Some choices of *C*(·,·) are given by Santner, Williams, and Notz (2003), which give varying degrees of smoothness. *C*(·,·) contains correlation length parameters, *δ*_*i*_, that need to be estimated (Liu & West, 2009; Vernon, Goldstein, & Bower, 2010; Williamson & Blaker, 2014).

Using an emulator, a prediction of the mean and variance for a parameter choice in X can be calculated, allowing uncertainty bounds to be placed on our predictions. Here, the focus will be on emulating functions that have a single output, although several approaches to emulating multivariate output exist, usually depending on the structure of the output (Bayarri *et al.*, 2007; Rougier, 2008; Higdon, Gattiker, Williams, & Rightley, 2008b; Liu & West, 2009; Conti & O'Hagan, 2010; Fricker, Oakley, & Urban, 2013; Sexton, Murphy, Collins, & Webb, 2011; Williamson & Blaker, 2014).

Using a Bayesian approach to fitting emulators and assuming the correlation parameters are fixed, prior knowledge about *f* can be represented by a Gaussian process (Haylock & O'Hagan 1996):

(2) $$f(\cdot )|\beta ,\sigma^{2}∼N(h{\left(\cdot \right)}^{T}\beta ,\sigma^{2}C(\cdot ,\cdot ))$$

where the scalar *σ* ^2^ is the variance. Assuming “non‐informative” prior distributions for the regression coefficients and variance, and given an ensemble *F* = (*f*(*x*_1_),...,*f*(*x*_*n*_)) of *n* runs of the computer model, a *t*‐distribution for *f*(*x*)|*F* is obtained, from which an estimate for the model output at *x* given the ensemble *F*, and the uncertainty on this prediction, can be calculated

(3) $$\frac{f(x)-m^{**}(x)}{\sqrt{\frac{F^{T}(A^{-1}-A^{-1}H{\left(H^{T}A^{-1}H\right)}^{-1}H^{T}A^{-1})FC^{**}(x,x)}{n-q-2}}}∼t_{n-q}$$

with posterior mean prediction

(4) $$m^{**}(x)=h{\left(x\right)}^{T}\hat{\beta }+t{\left(x\right)}^{T}A^{-1}(F-H\hat{\beta })$$

and posterior correlation between two points given by

(5) $$\begin{array}{cc} C^{**}(x,x^{\prime }) & =\;C(x,x^{\prime })-t{\left(x\right)}^{T}A^{-1}t(x^{\prime })+(h{\left(x\right)}^{T}\\ & \;-t{\left(x\right)}^{T}A^{-1}H){\left(H^{T}A^{-1}H\right)}^{-1}{\left(h{\left(x^{\prime }\right)}^{T}-t{\left(x^{\prime }\right)}^{T}A^{-1}H\right)}^{T}. \end{array}$$

where *H* is the design matrix with rank *q*, *A* is the correlation matrix with *i*,*j* ^*th*^ entry *C*(*x*_*i*_, *x*_*j*_), and *t*(*x*) is a vector of length *n* with *i* ^*th*^ entry *C*(*x*, *x*_*i*_).

In many applications, the correlated residual in the above formulation is not included, with instead a regression‐only approach used. This is equivalent to assuming *ε*(*x*) = 0 so that the whole residual is the nugget. This has particularly been the case in climate applications (Rougier, Sexton, Murphy, & Stainforth, 2009; Sexton, Murphy, Collins, & Webb, 2011; Holden *et al.*, 2013). It is computationally efficient to evaluate predictions and uncertainties using a regression model rather than a Gaussian process, as well as being easier to fit, so only fitting a regression can be an attractive option. However, an important question to consider is whether too much information is being lost by not having the correlated residual term, or whether any improvement is too negligible given the extra time and expertise required to fit a Gaussian process. We will measure the effect of these two approaches in the context of calibrating a computer model using HM.

### 2.2 History matching

History matching is a technique developed to rule out parameter settings of a computer model based on historical observations of the physical system (Craig, Goldstein, Seheult, & Smith, 1996; Vernon, Goldstein, & Bower, 2010; Williamson *et al.*, 2013). HM may be used as an alternative to BC, or as a step prior to this. It is a more flexible method than BC, as it is possible to decide to match on easy‐to‐model outputs first, removing nonphysical behavior, so that the modeling of other outputs may become more straightforward. A further benefit is that we only need to specify the means and variances of the quantities of interest (Craig, Goldstein, Seheult, & Smith, 1996), as opposed to the full distributions required in BC.

History matching is used to explore what the computer model is unable to do by ruling out regions of parameter space that are inconsistent with the observational data and a given uncertainty description. This can be done for any output of the model for which observational data is available. Where it is infeasible to run the computer model at millions of points in parameter space in a reasonable time frame, an emulator is required for all outputs of the model that we wish to use for HM, built using an available ensemble of runs of the computer model.

In order to use HM, a statistical relationship between the computer model *f*(·) and the underlying system it represents, *y*, is required. Kennedy and O'Hagan (2001) suggested

(6) $$y=f(x^{*})+\eta$$

where *η* is the discrepancy between the real world and the model representing it, and is independent of the model *f*(*x*). In reality, it is only possible to observe *z*, which is the true value of the real‐world system with some unknown error, *e*:

(7) z=y+e

where *e* has mean zero, and is independent of *y*.

In HM, we define the “implausibility” (Williamson *et al.* 2013) as:

(8) $$I(x)=\frac{\left|z-E[f(x)]\right|}{\sqrt{\mathrm{Var}[z-E[f(x)]]}}$$

where E[*f*(*x*)] is the prediction from an emulator. The denominator of the implausibility incorporates the variance of the measurement error *e* and the variance of the discrepancy *η*, as well as the variance on the prediction given at *x* by the emulator. Using (6), this expression is (Craig, Goldstein, Seheult, & Smith, 1996)

(9) Var[z−E[f(x)]]=Var[f(x)]+Var[e]+Var[η].

We then rule out parameter settings that are “sufficiently far” from the observations, in that it is unlikely that when *f*(·) is run at these parameter choices, it will give an output that is close to the observed output, given (6) and (7). “Sufficiently far” is defined using the implausibility, according to a user‐defined tolerance to error; often, 3 is used, based on Pukelsheim's Three Sigma Rule (Pukelsheim, 1994). The space that has not yet been ruled out is called “Not Ruled Out Yet (NROY)” space, and is where any future ensemble runs should be focused:

(10) $$X_{\mathrm{N ROY}}=\{x_{0}\in X|I(x_{0})<a\}$$

History matching should be done in several waves, as in Vernon, Goldstein, & Bower, 2010; Williamson, Blaker, & Sinha, 2016. In the first wave, an ensemble is designed to cover the whole input space, and an emulator is constructed based on these runs of the computer model. HM is then carried out as above. Assuming that NROY space is nonempty, a new ensemble of runs can be designed based on this NROY space, and a second wave of HM can be done after building an emulator for these new runs. This method is called “refocussing.” An advantage of refocussing is that it improves the accuracy of our emulators in the region we are most interested in (NROY space), as we have denser samples in this space as we progress to later waves. The new emulator that we build with this sample only needs to be accurate in NROY space; hence, the use of a stationary Gaussian process is more reasonable, and we should have a more representative proxy for *f*, leading to improved accuracy in HM.

This is an alternative to the method outlined by Gramacy *et al.* (2015), where Gaussian process emulators are fitted locally to an ensemble of 26,458 model runs. Refocussing is preferred for improving our emulators and HM due to our ensemble sizes being in the order of 10s or 100s in most examples, so the benefits of local fitting in the original large parameter space may not be substantial.

### 2.3 Motivation

Using a Gaussian process reduces the uncertainty around design points, but computer models have high‐dimensional input space, so these design points tend to be sparse. Gaussian processes were originally used in HM and BC, but due to the computational expense required to evaluate the Gaussian process often enough so that the implausibility space can be mapped, or so that MCMC can be performed to sample the posterior of *x* ^∗^, regression‐only emulators, where the residual variance is all nugget, have been used in many environmental applications (Rougier, Sexton, Murphy, & Stainforth, 2009; Edwards, Cameron, & Rougier, 2011; Sexton, Murphy, Collins, & Webb, 2011; Williamson *et al.*, 2013; Williamson, Blaker, Hampton, & Salter, 2015). Those papers that involve multi‐wave HM act on the assumption that by performing multiple waves, it is possible to fit regressions at the start to save computational time and that by fitting Gaussian processes at later waves, or by simply performing more waves, it is possible to achieve the same NROY space than if we were to only use Gaussian processes. This investigation looks into whether this is a valid assumption or if the effects of ignoring the correlation between outputs in the emulator are substantial.

There will be some improvements made by using Gaussian process emulators, but it is unclear whether this would be worth the extra time required to fit the model and then the extra computational time required to make the desired predictions. When multiple waves of HM are carried out, to determine whether a point is in NROY space, the emulator from each wave must be evaluated at this point. When this is done for millions of points in parameter space, as in HM, this could be burdensome if the number of waves is large and will take significantly longer than if we were to use regressions as our emulators. Therefore, whether we should use a Gaussian process at all, or when we should start to use a Gaussian process in a multi‐wave experiment, are important questions.

To assess the impact of including a Gaussian process in a high‐dimensional space, a 10‐dimensional toy function, described in Appendix A, was defined, and then a regression‐only and a Gaussian process emulator were fitted based on a sample from parameter space.

**Figure 1:**
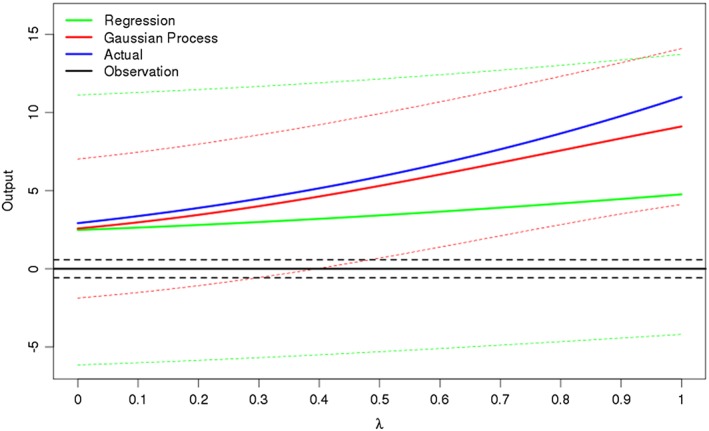
How the prediction and 99% uncertainty bounds change for a regression‐only emulator (green) and a Gaussian process emulator (red) for a line between two design points *x*_1_, *x*_2_ in 10‐dimensional space, where λ describes how far along this line we are. The actual function (blue) is a toy model. The Gaussian process is a better approximation of the original function and has less uncertainty on its predictions here. The observation is taken to be 0, observed with an observation error given by the dotted black lines

Figure 1 shows the predictions from these two emulators, along with 99% uncertainty bounds, on a line through 10‐dimensional space between two design points. It shows that the Gaussian process is a better approximation of the true function here and also that the uncertainty is less everywhere along this line. Furthermore, if we were to take 0 as our observation (with the dashed black lines representing the observation uncertainty and discrepancy), we can see that according to the regression emulator, we cannot rule out any of this portion of space, despite the fact that the actual function is reasonably far from this. However, using the Gaussian process emulator, the uncertainty shrinks on the right side of the picture, no longer including 0 within it; hence, we can now say that this part of space leads to output not consistent with the observation.

**Figure 2:**
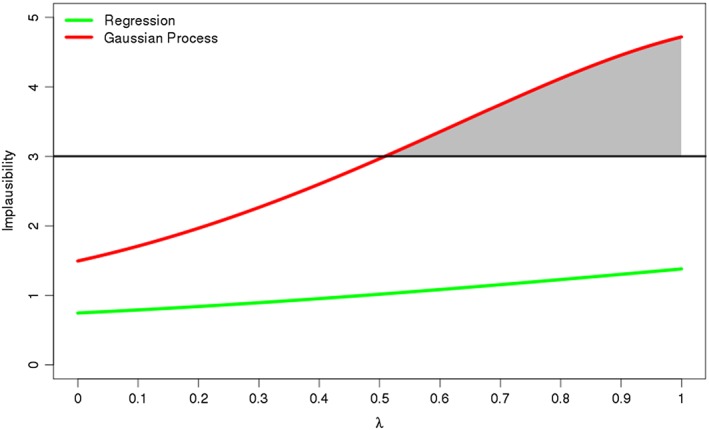
The implausibility I(x) for the above two emulators. With 3 chosen as the threshold for ruling out points, the regression emulator cannot rule out anything in this part of space, while the Gaussian process emulator can for λ > 0.52

How the implausibility changes along this line is illustrated in Figure 2. If we take 3 as the threshold above which we deem a run to be implausible, we see that the implausibility based on the regression emulator never passes this level, whereas the Gaussian process implausibility does.

#### 2.3.1 Tractability

On a standard desktop with four cores, it takes 1 s to evaluate a regression model at one million points, compared to 90 s for a Gaussian process (done in parallel), if n = 200. For n = 400, this calculation takes 2 s for a regression, compared to 188 for the Gaussian process. When the number of waves increases and the size of NROY space gets extremely small, as is the case in many climate applications, the difference in the time taken for these evaluations may become prohibitive.

In Lee *et al.* (2013), 8,192 independent emulators are built for the different grid boxes of the output of a global aerosol model. If these are all regression emulators, and we wish to find the model output for one million parameter choices, this calculation will take 8,192 s, or 2 hr and 15 min. If instead we have Gaussian process emulators (with n = 200), this will take 8.5 days.

Another example is Andrianakis *et al.* (2015), where nine waves of HM are performed, leading to an NROY space that is 10^11^ smaller than the original space. If we have Gaussian process emulators at each wave, this calculation will also be exponentially more expensive than the equivalent with regressions, as we will require millions of Gaussian process evaluations in order to build up a picture of an NROY space this small.

This motivates comparison of the two approaches in the full space. We will now explain the method that is used in order to make this comparison, with the results appearing in Section 3.

### 2.4 Methodology

Using each of our example functions, each time denoted f(*x*), we perform the following experiment:

1. An initial sample of size n is taken in parameter space, using a Latin hypercube maximin design (Morris & Mitchell 1995). The function is evaluated at these points, giving an ensemble F.
2. The ensemble is divided into training and validation sets. We fit a regression and a Gaussian process model to the training data. When fitting regressions and mean functions for the Gaussian processes, the maximum number of terms allowed is a 10th of the sample size. The correlation lengths and nugget are chosen using the algorithm in Appendix B. Before proceeding, diagnostic checks are carried out on these models.
3. The size of the NROY space defined by each of these emulators is estimated by taking uniform samples of 10,000 points in parameter space, until 1,000 points have been found that are not ruled out according to the chosen level of implausibility (here, this will be 3).
4. We repeat Steps 1–3 for a total of four waves of HM. At subsequent waves, we sample from the current NROY space instead of the entire parameter space to create the training set, with points from the previous wave that have not yet been ruled out used as the validation set.

In this case study, we will have an alternative multi‐wave experiment generated at each wave, as the result of taking an experiment that has thus far used regression only and now adds a Gaussian process within the current NROY space. This allows a direct comparison to the regression‐only approach, as the same sample is being used to fit the two different models. Additionally, all Gaussian process emulators define an NROY space from which a new Gaussian process emulator will be fitted, as we assume that once a Gaussian process has been used once, we will continue to use this method.

**Figure 3:**
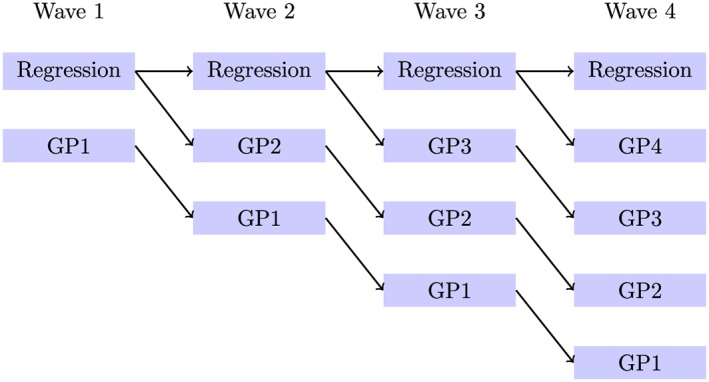
Flow chart showing the emulators built for a comparison between the regression‐only case and the Gaussian process case. GP1 denotes that we started to use a Gaussian process from Wave 1 in that history match

For example, at Wave 1, there are two emulators: the regression‐only emulator and the Gaussian process emulator. Then, at the second wave, there is a regression‐only emulator and a Gaussian process emulator that uses the Wave 1 regression to define NROY space, and there is a new Gaussian process emulator that uses the Wave 1 Gaussian process to define NROY space. Hence, at wave m, there are m + 1 emulators in each comparison: one where the regression‐only method is used, and m Gaussian process emulators, one that started from each of waves 1,...,m. In other words, there is one history match carried out using only regression‐only emulators, and history matches carried out separately by starting to use Gaussian process emulators from each wave (Figure 3).

Since the uncertainty shrinks around design points when we use a Gaussian process, we expect that we will rule out more space when history matching with a Gaussian process compared to using just a regression. Intuitively, one would expect that the more waves that the Gaussian process is used at, the smaller the resulting NROY space will be after four waves: using a Gaussian process at every wave should lead to the smallest space, followed by starting to use a Gaussian process at Wave 2, with the regression‐only progression giving the largest NROY space after Wave 4.

## 3 SIMULATION STUDY

We apply the methodology of Section 2.4 to four different test problems, each designed to have some of the features we regularly see when calibrating environmental simulators such as climate models. Function 1, given in Appendix A Equation (A1), represents a 10‐dimensional unknown function with no periods that should be well represented by a polynomial surface and where regression‐only emulation ought to perform well. Function 2 (A2) represents a more complex 10d function containing periodic functions that should favor the flexibility of the GP. Function 3 (A3) has an input space with 20 dimensions, the same size as the NEMO ocean model studied in Williamson, Blaker and Sinha (2016) to inform us as to whether local variability around extremely sparse points in high dimensions can influence calibration done in this way. Each of these three functions also contains some level of randomness, as each time we evaluate the function at a parameter setting *x*, we also sample from a normal distribution to add in some random noise; this is used to represent the internal variability in climate models. Finally, we also investigate the borehole function as it is a standard test problem (Worley 2015; Morris, Mitchell, & Ylvisaker, 1993) (A4).

A Bayesian regression approach is used, accounting for the uncertainty in the regression coefficients, since we do not know the true values of these. For the Gaussian process emulators, we use the squared exponential correlation function, with a nugget included in order to incorporate our knowledge that there is internal variability, by ensuring that our emulator will not interpolate the design points

(11) $$C(x,x^{\prime })=\nu I_{x=x^{\prime }}+(1-\nu )\exp\left\{-\underset{i}{\sum }\frac{{\left(x_{i}-x_{i}^{\prime }\right)}^{2}}{\delta_{i}}\right\}$$

where *I*_*x* = *x′*_ is 1 if *x* = *x* ^*′*^, and 0 otherwise.

The priors for the regression and variance parameters described in Section 2.1 are used here. We assume that the correlation lengths are fixed. When fitting a Gaussian process, we will attempt to explain as much of the response as possible with the mean function, as in Vernon, Goldstein, and Bower (2010).

For each toy function, four waves of HM has been carried out, following the method outlined above. For the first function, we have assumed that we have observed 15 (i.e., we want to find where in parameter space we can achieve this), with a measurement error variance of 10^−5^. The discrepancy variance is set to zero for each function. A random normally distributed noise (with mean 0 and variance 0.05^2^) has been added to the function output to represent initial condition uncertainty, as would typically be present in environmental models. For Functions 1, 2, and the borehole function, we have *n* = 200, and *n* = 400 for Function 3 (due to the higher dimension of X). The observations and measurement error variance for the other functions are given in Table 1.

**Table 1:** Function information for history matching. Range denotes the spread of possible outputs for the function, and Not Ruled Out Yet (NROY) size denotes the theoretical size of NROY space, given this error structure, and assuming a “perfect” emulator

Function	Range	z	Var[e]	Var[η]	Sample size	NROY size
1	[−42, 59]	15	10^−5^	0.05^2^	200	0.17%
2	[−3, 12.5]	9	10^−3^	0.15^2^	200	0.24%
3	[−145, 136]	50	10^−2^	0.5^2^	400	0.28%
Borehole	[0, 300]	100	10^−3^	0	200	0.11%

### 3.1 Size of NROY space

**Figure 4:**
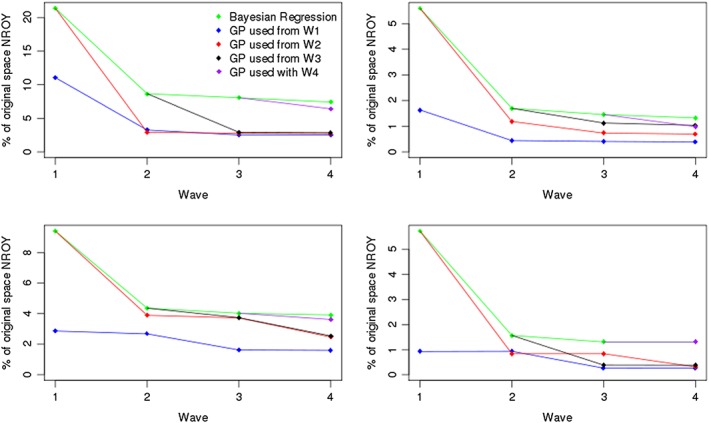
Top left Function 1, top right Function 2, bottom left Function 3, and bottom right borehole function. This picture shows the sizes of not ruled out yet (NROY) space we have at each wave when history matching our various functions with regression‐only emulators and when we start to use a Gaussian process emulator at different waves

The resulting sizes of the NROY spaces when history matching in Figure 4. Some of the exact percentages associated with these plots are provided in Table 2. We see that for each function, using a Gaussian process provides a large improvement over only using regressions. There is a reasonable difference at Wave 1 for each function, so even if only a single wave of HM is to be performed, as is often the case in applications, this suggests that a Gaussian process should be fitted. For example, for Function 1, using a regression emulator at Wave 1 gives an NROY space that is 21.38% of the original parameter space X. By using a Gaussian process instead, we are able to rule out nearly 10% more of the original space, leaving 11.05% of X as NROY, even though we expect a polynomial surface to do well here.

**Table 2:** The size of not ruled out yet (NROY) space (as a percentage of the original space) after Wave 4, for when only regression emulators have been used and for when a Gaussian process has always been used

Function	Regression	Gaussian process
1 (10d)	7.410%	2.525%
2 (10d)	1.326%	0.387%
3 (20d)	3.913%	1.596%
Borehole	1.308%	0.258%

It is a similar story for the other functions, with Function 2 giving Wave 1 NROY spaces with size 5.60% (regression) and 1.64% (Gaussian process), Function 3 giving 9.42% and 2.87%, and the borehole function 5.74% and 0.93% for the regression and Gaussian process, respectively. Furthermore, the NROY spaces defined by Gaussian processes after Wave 4 are much smaller than for the regression‐only approach, as shown in Table 2. For the borehole function, the NROY space after Wave 4 when Gaussian processes have always been used is less than 20% of the size when we use regressions.

We can now address the previously mentioned issue of whether using a Gaussian process is too computationally burdensome. For example, if we were to evaluate the mean and uncertainty for a million points in parameter space using a desktop computer, if we have a regression this takes 2 s, compared to over 2 min for a Gaussian process, for a 10‐dimensional function. However, we see that favorable results can still be achieved if a regression is used initially. For these examples, we do not always manage to find an NROY space that is extremely close to the “always Gaussian process” blue line (Figure 4) after four waves (i.e., for Functions 2 and 3, although the red line is still a good improvement over using regressions), but the pictures for Function 1 and the borehole function exhibit some convergence between the blue, red, and black lines by Wave 4, i.e., the size of NROY space at Wave 4 is similar, regardless of whether we fit a correlated residual term at Wave 1 or 2.

This is an extremely beneficial property, allowing computational savings to be made. This suggests that using a regression at the first couple of waves, before using a Gaussian process later on, may be a reasonable compromise, if computing time is an issue, and may be the best combination in terms of minimizing computation time while finding a reasonable NROY space. The regression would essentially be used to capture global variation in the function output, removing parameter settings based on this, before the Gaussian process is then used to model the local variability in our (possibly small) NROY space. Being able to focus an ensemble in a smaller NROY space will help with the accuracy of the Gaussian process emulator, as there will be a greater density of points, so the shrinking of variance around observed points will have a more profound effect than in the original space.

However, Figure 4 also highlights a discrepancy with our expectation of what the results will be: HM using a Gaussian process emulator more often has not always led to a smaller NROY space. At Wave 2 for Function 1, we find a smaller NROY space by using a regression at Wave 1 followed by a Gaussian process at Wave 2, than by using a Gaussian process at both waves, although the difference here is reasonably small. Starting to use a Gaussian process from Wave 4 does not have as large an effect as we might expect. We rule out more space than the regression does here, but every other time we started to use a Gaussian process from an NROY space defined solely by regressions for this function, we found a much larger impact by the Gaussian process.

For the borehole function, using a Gaussian process at Wave 2 having used one at Wave 1 fails to rule out any extra space (GP1/blue line). Furthermore, using a Gaussian process at Wave 3, having started to use Gaussian processes from Wave 2 (GP2/red) makes no difference. The same is true when we start to use a Gaussian process at Wave 4 (GP4/purple). This is not because all of the points yet to be ruled out are close to the observed value, as for both GP1 and GP2, we rule out more space at the next wave. This suggests that we have either chosen the correlation lengths for our Gaussian process poorly, or that we had a “bad” (in some sense) sample at this wave. This unexpected result is explored in Section D.

### 3.2 Composition of NROY space

Simply ruling out more space may not be desirable if our emulators are incorrectly ruling out points that are in fact close to the observations or are leaving regions of space that give output far from the observations. To show the composition of the NROY spaces that four waves of HM has produced, we now sample from various NROY spaces as defined above and look at a weighted density of the function outputs at these points, weighted by $e^{-I(x)}$. This has been chosen as the weighting function as points in parameter space with smaller values of I are more likely to be consistent with the observations. If our emulators are giving low implausibilities to points far from the observations, we want this to be accounted for in our analysis.

Note that for a uniform prior on the best input, and having made a best input assumption and all other normality assumptions given in Kennedy and O'Hagan (2001), the likelihood of the observations z is $e^{-I(x)}$ in NROY space and our re‐weighted sample might be considered to be a sample from the posterior distribution p(*x* ^∗^|z,F_1_,F_2_,F_3_,F_4_), assuming zero likelihood at points ruled out in previous waves.

**Figure 5:**
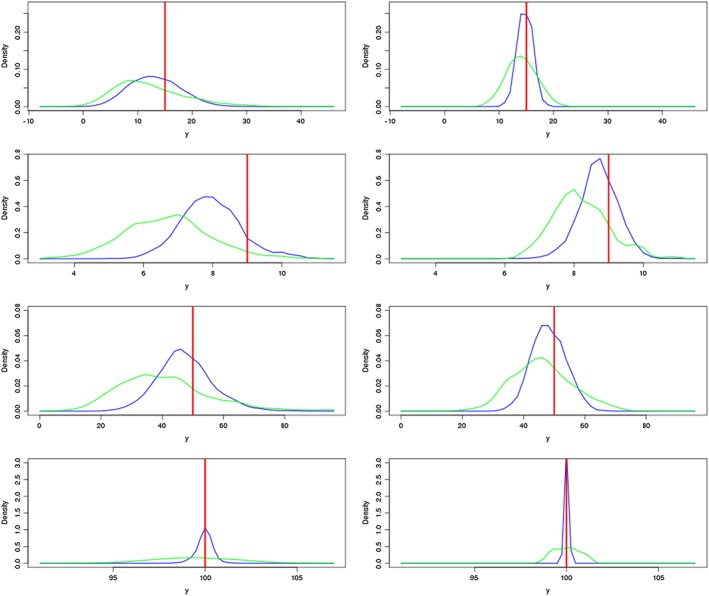
The weighted densities for the function output at points in NROY space after Wave 1 (left) and Wave 4 (right) for each of the four functions, for the Gaussian process (blue) and the regression‐only emulator (green). The observation for each function is given by the red line

Figure 5 shows these weighted densities for the NROY spaces defined after Waves 1 and 4 for each of our functions. The Wave 4 comparison is between the case where only regression is used, and where a Gaussian process is used at all four waves. We see that when a Gaussian process emulator has been used, the spread of outputs we are left with in NROY space, and hence, our parametric uncertainty, is decreased: when we use a Gaussian process, we are better at ruling out extreme values than when using a regression. This is the case after a single wave and after four waves. We would expect that after multiple waves of HM, the spread of the outputs in NROY space will have decreased, and this is shown here. For each function except the first, we have less residual parametric uncertainty after one wave of using a Gaussian process than we do after four waves of regression.

We can quantify the difference between the spread of outputs for the two emulators by looking at the variance. For Function 1, after the first wave the variance of outputs in our sample of points in NROY space is 43.45 when we use the regression emulator to define NROY space, compared to 26.98 for the Gaussian process NROY space. After Wave 4, the regression‐only approach has shrunk the variance to 11.19, but four waves of Gaussian process emulators gives an NROY space with a variance of 2.38 on the outputs.

We also observe that for every function, there is more weight around the true observation for the Gaussian process NROY space, both after one wave and four waves. For Function 1 after Wave 1, the regression density is skewed away from the observation, while the Gaussian process density is much closer to the observation. At Wave 4, both methods lead to an NROY space with greatest weight distributed closer to the true observations but with a far greater density for the Gaussian process.

Both densities exhibit bias for Function 2, but the Gaussian process assigns more weight closer to the observation. There is still a skew after four waves for the Gaussian process but less than for the regression. Function 3 is similar, in that we do not have any densities distributed around the observations, but there is an improvement between waves.

For the borehole function, the difference between the two emulator types is most pronounced. After the first wave, the majority of the density in the Gaussian process NROY space is around the observation, while the regression has similar weight for a wider range of outputs. By Wave 4, the regression is doing better, but the Gaussian process is still far superior, with the majority of the density within 0.25 of the observation. The Gaussian process approach clearly outperforms our regression emulator here.

In general, at the first wave, we have not only been able to remove extreme outputs more efficiently but also we have been able to assign more weight to the regions of parameter space that actually give observations closer to the true output, both of which will have an effect on inferences we make about the real system. This is important as when we want to take new samples in our existing NROY space, if the space of possible outputs is smaller, then we are more likely to find actual runs of the computer model close to our observations, which in turn allows us to more accurately emulate model behavior in this important region of parameter space.

## 4 THE IC FAULT MODEL

We now check whether our findings are consistent when we have an actual physical model that we are attempting to history match. The IC fault model is a cross‐sectional model of a reservoir, with three unknown parameters *h* (the fault throw), *k*_*g*_ (the good‐quality sand permeability), and *k*_*h*_(the poor‐quality sand permeability) (Tavassoli, Carter, & King, 2004; Tavassoli, Carter, & King, 2005). The IC fault model is a difficult function to accurately calibrate; hence, we are interested in attempting to history match instead. This is a good example of the types of environmental models that we may wish to history match as we have multiple outputs (although there is no internal variability).

**Figure 6:**
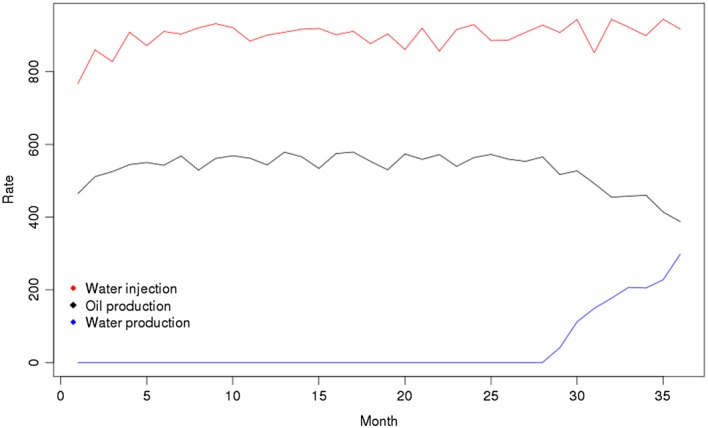
The observations for the IC fault model

The output at each parameter choice is a time series (of length 36 months) of the oil production rate, the water injection rate, and the water cut (or production) rate. We have a database of 159,661 runs of the model at different parameter choices. However, as we typically will not have access to this number of runs, we assume that we have not run the model at all of these parameter choices, and instead sample from our database in order to create an ensemble with *n* = 60. The observations that we wish to match to are given in Figure 6.

In the previous examples, we had a single output for each function, whereas here we have multiple outputs in the form of three time series. Instead of emulating each of these time series completely, due to correlations between the monthly values for some of the outputs, we history match using the following three statistics: o_24_, the oil production rate in Month 24; o_36_, the oil production rate in Month 36; and w_36_, the water injection rate in Month 36.

In order to history match, we need to know the observations. For the above three statistics, these are

(12) $$\text{z}={\left(563.6,387.5,917.2\right)}^{T}$$

Previously, we ruled out a parameter choice *x* if the implausibility at that point was greater than 3. Now, we will have a value for the implausibility for each of our three statistics, so we instead use the Second Maximum Implausibility Measure (Vernon, Goldstein, & Bower, 2010):

(13) $$I_{2M}(x)=\underset{i}{\max}\left(\left\{I_{i}(x)\}\underset{j}{\max}I_{j}(x\right)\right)$$

and then rule out a parameter setting *x* if this is greater than 3.

We set the observation error variance as 1 for each statistic, and the discrepancy variance as 0, as we are assuming that the model has parameter settings that can reproduce the observations, up to some measurement or observation error.

### 4.1 Results

**Figure 7:**
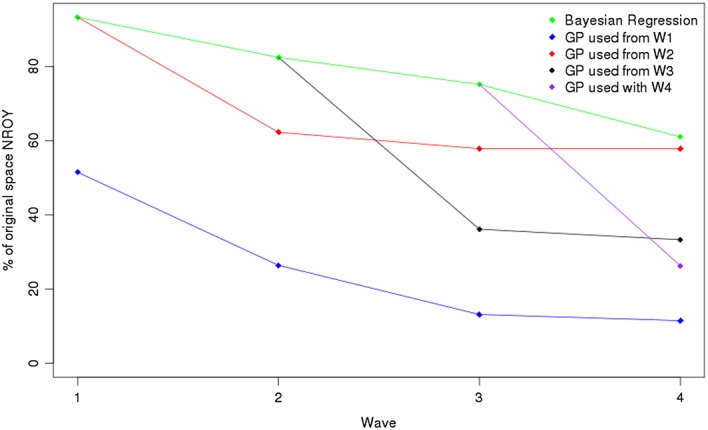
The progression of the sizes of NROY space when history matching the IC fault model with regression‐only emulators and when we start to use a Gaussian process emulator at different waves

As for the toy examples, we perform four waves of HM, with the comparison of regression and Gaussian process emulators as before. The results are shown in Figure 7.

As in the previous examples, we see that the Gaussian process cases all outperform the regression‐only history match: if we use a Gaussian process at every wave, we find an NROY space that is 11.5% of the original parameter space, compared to 61% if we were to only use regressions. We also observe a large improvement if we use regressions for the first three waves followed by a Gaussian process at Wave 4, giving an NROY space of size 26.3%.

**Figure 8:**
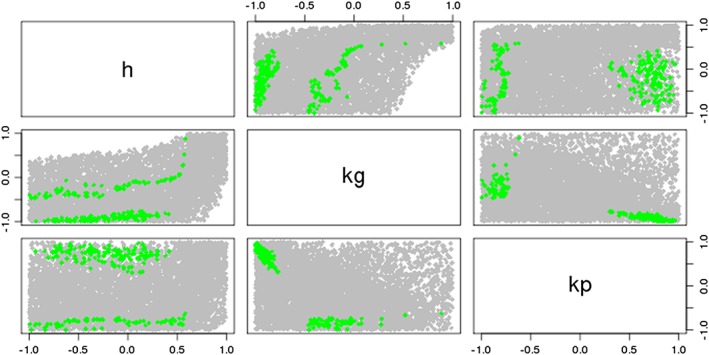
A parameter plot showing the true NROY space (green) and those points classified as being in NROY space after four waves when we use regressions at each of the four waves

**Figure 9:**
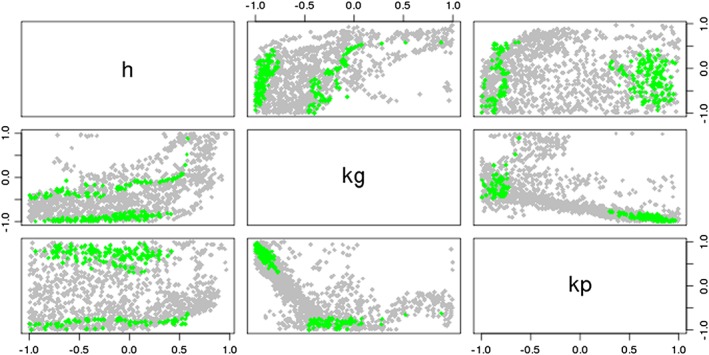
A parameter plot showing the true NROY space (green) and those points classified as being in NROY space after four waves when we use Gaussian process emulators at each of the four waves

In Figures 8 and 9, we see that the true NROY space for the IC fault model is split into two disjoint regions of the parameter space (as shown in Tavassoli, Carter, & King, 2005). Figure 8 shows that when we use the regression‐only emulators, we are only able to rule out some of the edges of parameter space, as shown by the white parts of the plots. When we use a Gaussian process at each wave, as for Figure 9, although we have not been able to find the two disjoint parts of NROY space, we have ruled out a lot more of the space around these, while also keeping the truth as part of our solution. We see from this that not only have we been able to rule out more space using the Gaussian process emulators, but we are also beginning to see the structure of the true NROY space, while the two‐dimensional parameter plots for the NROY space defined by using regressions at each wave do not tell us very much about the composition of the true NROY space.

We once again observe the importance of the design of the sample, as we have been unable to rule out much additional space after Wave 2 when we use a regression at Wave 1 followed by a Gaussian process thereafter. This does still give superior results to four waves of regression. A reason for the sampling problem here may be due to the composition of the true NROY space, which may lead to difficulty in emulating the outputs in this manner.

If we were to continue to history match this model by running more waves from this starting point, in order to find the true NROY space, we would need to build separate emulators for these different regions of parameter space, as the behavior of the output in the disjoint regions of NROY space may be different. Additionally, as we have access to three time series of model output, we should add in more uncorrelated outputs in order to remove space more efficiently.

## 5 DISCUSSION

We have compared the statistical emulation techniques of regression and Gaussian processes in the context of multi‐wave HM as a way of quantifying parametric uncertainty. We have used two 10‐dimensional toy functions, a 20‐dimensional toy function, and the borehole function in order to compare the sizes of NROY spaces that result from using various combinations of regressions and Gaussian processes at different waves. We have then suggested reasons why we may sometimes observe a premature convergence in the size of NROY space. We have then seen that we reach the same conclusions if we use an actual physical model instead of toy examples.

The results outlined above suggest that it is beneficial to use a Gaussian process emulator when history matching, either in a single wave or multiple waves. When performing a single wave, using a Gaussian process has been found to offer a significant improvement both in terms of the size and the composition of the resulting NROY space to a regression emulator, therefore having a substantial impact on any inference we make about the real system. Performing a single wave has always been the practice in BC‐only analyses (Kennedy & O'Hagan, 2001; Rougier, 2007; Higdon, Nakhleh, Gattiker, & Williams, 2008a; Sexton, Murphy, Collins, & Webb, 2011), so using a Gaussian process (as advocated by Kennedy & O'Hagan 2001) rather than regression (for example, Sexton, Murphy, Collins, & Webb 2011) should allow inferences to be improved.

In many applications (especially climate), regression‐only emulators are used (Rougier, Sexton, Murphy, & Stainforth, 2009; Sexton, Murphy, Collins, & Webb, 2011; Holden *et al.*, 2013), with the expectation that fitting a Gaussian process makes little difference to the calibration or HM, due to the huge parameter spaces and small n. However, we have shown that even in this setting, the cumulative effect of the variance shrinkage around these sparse points is enough to have a significant and lasting effect on the analysis. Over multiple waves, using a Gaussian process adds up to a large improvement over the regression‐only case, and in some cases, it appears to be difficult to make up the difference by performing more waves of regression. Due to the long computational times and large numbers of emulators or waves required (Section 2.3.1), there is perhaps some trade‐off required between the two types.

Therefore, where it is possible to carry out HM in multiple waves (which should be done whenever available resources allow it), using a regression emulator to rule out space at the first wave may be acceptable. This allows space to be ruled out based on global behavior initially, before looking in more detail at local behavior by fitting a Gaussian process at later waves. This is a reasonable approach as some convergence between the size and composition of NROY spaces defined by starting to use Gaussian processes at different waves has been observed, which would allow computational time to be saved thanks to requiring fewer Gaussian process emulators to be evaluated at millions of points in parameter space, as well as reducing the need for fitting correlation lengths and the nugget, which can be difficult and time‐consuming.

In our final example, the IC fault model, we have multiple outputs as would often be the case in HM and have found the same results as for our idealized examples, with the Gaussian process cases outperforming the case where we only used regressions. We have seen the importance of ensemble design, and have also highlighted that if we were to history match this model further, we should get superior results by building separate emulators for different regions of parameter space due to the nature of NROY space.

## Data Accessibility

Data pertaining to this manuscript is deposited in figshare at DOI: http://dx.doi.org/10.6084/m9.figshare.3474293

- Data File 1: Paper final env after review.bbl – List of references

## Figures and Tables

**Figure C1:**
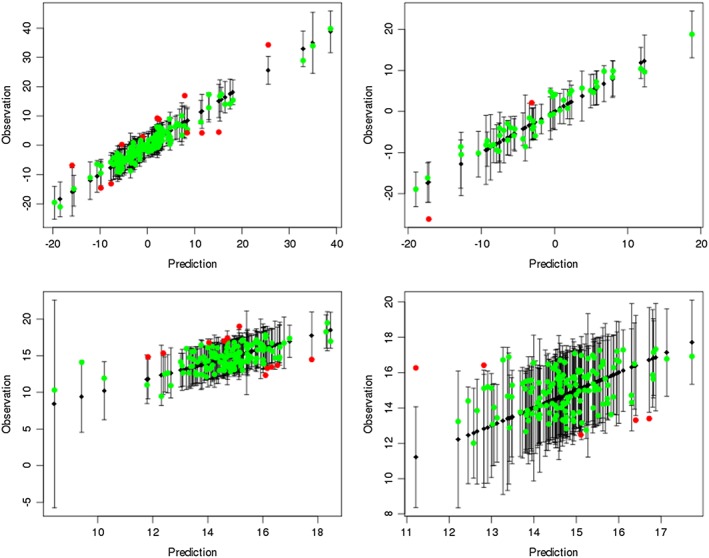
Leave‐one‐out cross validation plots (left) and prediction for the validation set (right), for the Gaussian process emulators for Function 1, after Wave 1 (top) and Wave 4 (bottom). The black points indicate the prediction given by the emulator, with 95% error bars. The green and red points are the actual function values, colored green if they lie within the 95% error bars around the prediction. Emulators are deemed to validate well if there are not too many or too few of the true values outside of these error bars. These checks ensure that the parameter estimation for the Gaussian process is reasonable and that our emulator has predictive power

**Figure D1:**
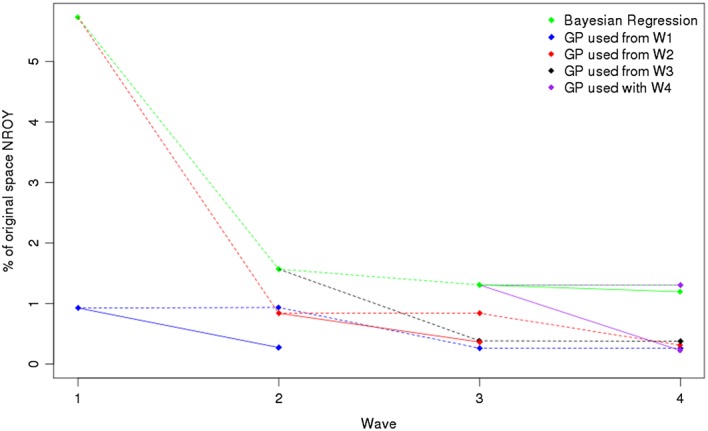
The progression of the sizes of NROY space for regression and the Gaussian processes for the borehole function. The dotted lines indicate the original NROY spaces found, as in Figure 4, with the solid lines showing improvements we have been able to achieve through either fitting a new mean function (in the case of GP2 [red line]), or by taking a new sample in the existing NROY space

## APPENDIX 1

### 1.1 A. Functions

*Ψ*(0,σ ^2^) denotes a random draw from a normal distribution with mean 0 and variance σ ^2^. Function 1 (10d):

(A1) $$\begin{array}{cc} f_{1}(x) & =7(x_{1}-1)+10(x_{1}+1)x_{2}{\left(x_{3}+0.5\right)}^{2}+5x_{4}^{2}\\ \;+5\exp(x_{5}(x_{6}+0.5))+x_{7}^{2}x_{8}^{3}+0.5x_{9}x_{10}\\ \;+0.5x_{7}x_{10}^{2}+2x_{5}x_{8}^{2}+\Psi (0,0.05^{2}) \end{array}$$

Function 2 (10d):

(A2) $$\begin{array}{cc} f_{2}(x) & =\sin(x_{1}x_{2})+\cos(x_{3})\sin(x_{4})\cos(x_{5}+x_{6})\\ \;+\sin(x_{1}x_{7})\cos(x_{7}x_{8})\exp(x_{9}+x_{3})\\ \;+\sin(\cos(x_{10}+x_{5}+x_{8}))\cos\left(x_{9}^{2}x_{3}\right)\\ \;+\sin(x_{7})+\cos(\sin(x_{5}+x_{9}))\\ \;+\exp(\sin(x_{2}\sin(x_{10})))\\ \;+x_{7}^{2}\cos(x_{1})\cos(x_{3})\\ \;+\exp\left(x_{2}^{2}\right)+\cos(x_{1}-x_{6})\\ \;+\Psi (0,0.15^{2}) \end{array}$$

Function 3 (20d):

(A3) $$\begin{array}{cc} f_{3}(x) & =5x_{1}(x_{2}{\left(x_{3}+0.5\right)}^{2}x_{4}+5\exp(x_{3}(x_{6}+0.5))\\ \;+x_{7}^{2}x_{8}^{3}+1.5x_{9}x_{10})+x_{5}^{2}+6\left(x_{11}+x_{12}^{3}\right)\\ \;+0.5(x_{12}-x_{13}x_{14})+x_{5}\exp(x_{15})\\ \;-10\exp(x_{16})\left(x_{17}+x_{18}+x_{19}^{2}+x_{20}^{3}\right)\\ \;+\Psi (0,0.5^{2}) \end{array}$$

Borehole function:

(A4) $$f(T_{u},H_{u},H_{l},r,r_{w},L,K_{w},T_{l})=\frac{2\pi T_{u}(H_{u}-H_{l})}{\ln(r/r_{w})\left(1+\frac{2LT_{u}}{\ln(r/r_{w})r_{w}^{2}K_{w}}+\frac{T_{u}}{T_{l}}\right)}$$

### 1.2 B. Fitting Gaussian Process parameters

First, an initial guess for the values of the correlation length parameters, *δ*, and the nugget, *ν*, needs to be made. This can be done using a heuristic, such as the one outlined in Vernon *et al.* (2010), where the initial values for the parameters are based on the order of each term in the regression model. At later waves, a sensible starting point would be the parameter values from the previous wave. We select the parameters after dividing the ensemble into a training set and a validation set, fitting them only based on the training set, and then checking that these choices lead to adequate predictions for the runs in the validation set. We perform leave‐one‐out cross‐validation based on the training set and seek to minimize the average standard error on our predictions while roughly 95% of the true values lie within the prediction error bars. We also predict the output for the runs in the validation set in order to assess the predictive qualities of the emulator, requiring that “enough” of the true values are within the error bars. Using these statistics, we can search for the best values for these parameters using the following algorithm:

1. Choose initial values for *δ*, *ν*.
2. For *i* = 1,…, *p*, allow the value of *δ*_*i*_ to change by 0.1.
3. Fit an emulator using these new parameter values.
4. Calculate the average standard error when performing leave‐one‐out cross‐validation, and count the number of predictions that lie outside 95% error bars for the cross‐validation, and when predicting the output for the validation set.
5. If this parameter setting is an improvement, return to Step 2, starting from the current values of *δ* and *ν*. Else set *i* = *i* + 1, and return to Step 2.
6. When *i* = *p* + 1, allow the value of *ν* to change by 0.0005, and proceed as for the *δ*_*i*_s.
7. Once all parameters have been allowed to change, pick the settings that minimize our statistic while not having too many or too few cross‐validation or test set predictions lying outside the 95% error bars.

0.1 has been chosen as the amount parameters are varied by at each step as, from experience of fitting these types of emulators, it was found that this gives a reasonable compromise between the ability to find good choices of the correlation lengths and the amount of computing time required to fit emulators for each different parameter choice.

This method may not lead to parameter settings that are strictly optimal, but it does allow us to find values that are consistent with the sample data and does so automatically in short enough of a time frame, so that we are able to build the large number of emulators required for this study. The maximum likelihood approach is generally outperformed by this method in our examples and was not satisfactory in some situations, hence the choice of an alternative way of selecting the correlation lengths. It is possible to use simulated annealing as a means of minimizing our statistic; however, this takes significantly longer to find a good setting of the parameters. Our algorithm offers the desirable combination of finding acceptable correlation lengths while not taking an excessively long time to do so.

The emulators that we have automatically fit using this method have been found to have good predictive abilities and offer a large improvement over using only a regression. We expect that if we only wished to fit a few emulators, we could achieve improvements at least as large by taking more time over the fitting of the correlation lengths.

### 1.3 C. Emulator validation

Due to the large number of emulators that have been required for this study, it has not been possible to fit the correlation lengths or check validation plots for all of these by hand. Some validation plots are given in Figure C1.

### 1.4 D. Sensitivity to sample design

We highlighted above that some of the results shown do not correspond to what we might expect. We focus on the case of the borehole function here where a Gaussian process is used from Wave 2 onwards, since there is little difference in the size of NROY space at Waves 2 and 3 when we performed our experiment. This was unexpected, given that at Wave 4 we are able to rule out additional space, so the reason for the leveling off after Wave 2 is not that it is no longer possible to rule out any further parts of parameter space. Additionally, by using just a regression for the first two waves, followed by a Gaussian process at Wave 3, we find a smaller NROY space than when we used a Gaussian process from Wave 2. The reason for this unexpected result is likely to be due to the sample we use or the estimation of the correlation lengths for our emulator. We now attempt to “fix” the leveling off in the size of NROY space shown here.

Refitting the correlation lengths and nugget from alternative starting points while still using the original sample was unable to offer much or any improvement. In order to investigate what is happening, additional samples were taken from NROY space, and emulators fitted to these, and the size of the new NROY space estimated. This allowed us to find a “best” sample, in the sense that this led to the smallest NROY space. From this, we have a new mean function and estimates of the correlation lengths and nugget.

We can fit this “best” emulator to our original sample. Doing so gives a smaller NROY space, of a similar size to that found using our best sample. This suggests that the problem is in fitting the emulator: choosing the mean function and then estimating the correlation lengths. Since with a poor mean function, we were unable to find correlation lengths that led to a smaller NROY space, we believe that if we can choose the mean function well enough, then we should be able to find reasonable correlation lengths. This in turn implies that the problem is in the design of the sample: when we have a different sample, we can fit a better mean function, and hence, emulator, whereas our automatic method for fitting emulators did not find this when it had access to the original sample.

In our automatic approach for fitting large numbers of emulators, the mean function is selected via a stepwise regression approach, with a random noise variable included as one of the predictors. Once the noise variable is selected by the stepwise algorithm, we add no further terms. The noise variable is a random sample from a zero mean normal distribution and has the potential to be correlated with other inputs. We can also end up selecting different mean functions given different random noise. We attempt to account for this problem by sampling the noise 10 times, and fitting a new mean function each time, then selecting the best mean function from these. This can still lead to poor choices, however, and this is what has happened in the borehole case above. In the 10 samples, the best mean function had only seven terms and highlighted only three of the eight variables as active.

By taking further samples of the noise, we can find a mean function with more terms in it that uses all of the borehole function parameters. The majority of the time we take a new sample of the noise, we end up with a poor mean function. If we now fit a Gaussian process emulator with our new mean function, we find an NROY space of size 0.3641% instead of 0.8392%. Figure D1 shows the NROY size progression for the borehole function, with this new emulator added. This looks more in line with what we expected to happen and with the results from the other functions. This demonstrates that selection of the mean function is important. As our automatic approach struggled to find good mean functions, despite taking several different noise samples, it also suggests that the sample design is crucial, as this directly affects the emulators we can fit. When we have taken alternative samples from our NROY spaces, it has often been much easier to find a good emulator: in many cases, the first mean function we fit leads to an emulator that gives a smaller NROY space. If we have a better sample, it is much easier to fit a good model. What “better” is in this case is an open question. As we sometimes struggled to select a good mean function for samples, it is not inconceivable that sometimes we will not be able to improve the perceived anomalies in the sizes of the NROY spaces we find.

Indeed, we are not able to solve the other problems highlighted in Section 3.1 by fitting a new mean function, suggesting that we may have a “bad” design in our existing NROY spaces for these waves. This may be down to not placing a design point in a part of space that turns out to be important, and it is, perhaps, impossible to account for that in any design methodology. Another reason we may have trouble emulating these functions in our NROY spaces could be if an NROY space were not connected. Such a problem would be very difficult to detect in any application and would lead to the fitting of one poor emulator (e.g., because high‐leverage points were used in the fitting of the mean function), instead of the ideal separate emulator for each separate region of space.

In summary, the leveling off at Wave 2 by the “always Gaussian process” case has been corrected by taking a new sample from the Wave 1 NROY space and then fitting a new Gaussian process emulator to this. The leveling off at Wave 4 when we start to use a Gaussian process after three waves of regression is also dealt with in this manner. We may expect that adding a correlated residual term should be able to make up for a poor choice of mean function. Indeed, this is part of the reason that many papers advocate a constant mean (Sacks *et al.* 1989a). The fact that in numerous applications of the method, we found this not to be the case should be troubling. A possible reason for this finding may be that the data are not well approximated enough by the weakly stationary processes we have been seeking to fit to them, even though the emulators each pass the standard validation tests.
